# Techno-economic Analysis of Sustainable Biofuels for Marine Transportation

**DOI:** 10.1021/acs.est.2c03960

**Published:** 2022-11-21

**Authors:** Shuyun Li, Eric C. D. Tan, Abhijit Dutta, Lesley J. Snowden-Swan, Michael R. Thorson, Karthikeyan K. Ramasamy, Andrew W. Bartling, Robert Brasington, Michael D. Kass, George G. Zaimes, Troy R. Hawkins

**Affiliations:** † Pacific Northwest National Laboratory, Richland, Washington99352, United States; ‡ National Renewable Energy Laboratory, Golden, Colorado80401, United States; § Oak Ridge National Laboratory, Oak Ridge, Tennessee37830, United States; ∥ Argonne National Laboratory, Lemont, Illinois60439, United States

**Keywords:** heavy fuel oil, marine biofuels, decarbonization, techno-economic analysis, sustainability

## Abstract

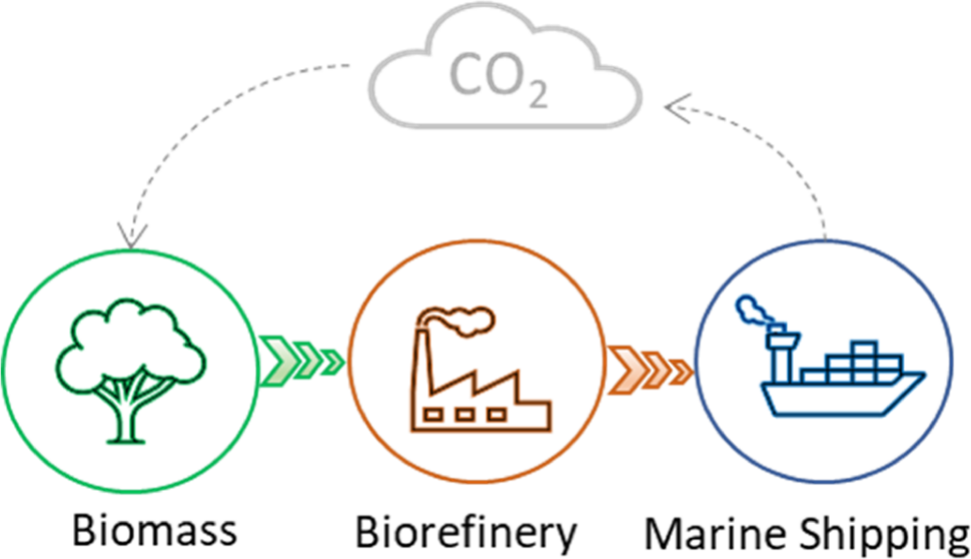

Renewable, low-carbon biofuels offer the potential opportunity to decarbonize marine transportation. This paper presents a comparative techno-economic analysis and process sustainability assessment of four conversion pathways: (1) hydrothermal liquefaction (HTL) of wet wastes such as sewage sludge and manure; (2) fast pyrolysis of woody biomass; (3) landfill gas Fischer–Tropsch synthesis; and (4) lignin–ethanol oil from the lignocellulosic ethanol biorefinery utilizing reductive catalytic fractionation. These alternative marine biofuels have a modeled minimum fuel selling price between $1.68 and $3.98 per heavy fuel oil gallon equivalent in 2016 U.S. dollars based on a mature plant assessment. The selected pathways also exhibit good process sustainability performance in terms of water intensity compared to the petroleum refineries. Further, the O and S contents of the biofuels vary widely. While the non-HTL biofuels exhibit negligible S content, the raw biocrudes *via* HTL pathways from sludge and manure show relatively high S contents (>0.5 wt %). Partial or full hydrotreatment can effectively lower the biocrude S content. Additionally, co-feeding with other low-sulfur wet wastes such as food waste can provide another option to produce raw biocrude with lower S content to meet the target with further hydrotreatment. This study indicates that biofuels could be a cost-effective fuel option for the marine sector. Marine biofuels derived from various feedstocks and conversion technologies could mitigate marine biofuel adoption risk in terms of feedstock availability and biorefinery economics.

## Abstract 2

Biofuels are promising options to replace existing fossil marine fuels to meet emission targets and mitigate climate change without significant changes to the maritime sector infrastructure. This study reports the economic feasibility and process sustainability of selected alternative marine biofuels to displace conventional marine fossil fuels.

## 1 Introduction

Industrial shipping is the backbone of the global economy and accounts for more than 90% of the world trade.^1^ The global shipping demand is expected to grow due to the expanding global supply chain, increasing population, and growing economy. The total annual energy requirement for the marine shipping sector is expected to increase from 10.5 to 24.5 exajoules by 2050.^2^ Currently, about 77% of the energy consumed for the propulsion of ships comes from the low-cost and abundant heavy fuel oil (HFO), a residual byproduct of crude distillation and cracking units.^3^ The HFO fuel contains polycyclic aromatics, high sulfur, heavy metals, and other impurities. Consequently, the combustion of marine HFO emits a wide variety of pollutants, including sulfur oxides (SO_*x*_), nitrogen oxides (NO_*x*_), particulate matter (PM), and carbon dioxide (CO_2_), which account for 14 to 31, 4 to 9, and 3 to 6% of global NO_*x*_, SO_*x*_, and CO_2_ emissions, respectively.^4^

The marine sector is aware of the need for clean, sustainable fuel. Accordingly, the International Maritime Organization (IMO) has set future targets for reducing the SO_*x*_, NO_*x*_, and carbon emissions from shipping. These include (1) a reduction in carbon intensity for international shipping by at least 40% by 2030; (2) a reduction of 70% carbon emissions by 2050 compared with the 2008 baseline; and (3) a maximum sulfur content of 0.5% in marine fuel or installation of scrubbers by 2020.^5^ To achieve the low sulfur requirement and lower carbon emissions, alternative marine fuels and new technologies are considered the most viable options in the near and long terms. Among the available options, the maritime community has identified that biofuels, liquefied natural gas (LNG), methanol, hydrogen, and ammonia are the most promising solutions. At the same time, new technologies such as battery systems, fuel cells, and wind-assisted propulsion can offer the potential for improving the modern vessel efficiency in the long term.^2^ There is great interest in using LNG as a marine fuel, and the number of ships powered by LNG is growing rapidly. While LNG can effectively reduce SO_*x*_, NO_*x*_, and PM emissions, there are concerns about its effectiveness to reduce greenhouse gas (GHG) emissions and the limited available infrastructure and bunkering facilities to support LNG as the shipping fuel.^6^ Ammonia and hydrogen have been considered potential pathways to net-zero carbon emissions as these carbon-free molecules can be produced from renewable energy sources. The main barriers are the safety issues for handling ammonia and hydrogen systems, respective new engine and fuel cell development, and economic feasibility.^7^ More detailed assessments are needed to understand the potential of ammonia and hydrogen as alternative marine fuels at scale.

Biofuels are fuels produced from biomass materials and are among the most promising options to replace the existing fossil marine fuels to meet the IMO emission targets before the middle of the century without significant changes to the existing maritime sector infrastructure.^8^ Biofuels have been demonstrated to reduce the net carbon emissions due to the uptake of carbon from the atmosphere during the biomass growth and play an essential role as a future marine fuel that is more renewable.^5^ Additionally, biofuels generally exhibit low to zero sulfur content. Therefore, the produced low-sulfur biofuels from carbon-neutral residual biomass and the low-cost wet waste biomass (*e.g.*, sewage sludge and manure) can meet CO_2_ emission reduction as well as the stringent sulfur requirements. Another advantage of biofuels for marine applications is that they can be used as a ready, drop-in fuel with minor changes required for the existing ship engines and infrastructure such as bunkering vessels. Several biofuels produced *via* various pathways, such as fermentation,^9^ pyrolysis,^10,11^ Fischer–Tropsch (FT) synthesis,^12^ and hydrothermal liquefaction (HTL),^13,14^ are being investigated for their compatibility with the current ship engines and infrastructures. Furthermore, BP has partnered with Maersk Tankers to test the biofuel derived from 30% fatty acid methyl esters blended with very low sulfur fuel oil (VLSFO) on the vessels sailing from Rotterdam to West Africa in 2021.^15^ Stathatou *et al.*(16) tested onboard emissions and well-to-wheel life cycle emissions using a 50:50 biofuel blend of used cooking oil biodiesel and marine gas oil on a two-stroke marine diesel engine of a Kamsarmax vessel. Such onboard measurements under actual trials provide critical information on the emission inventories, engine operation, and performance.

Even though pathways to produce marine biofuels have been demonstrated, there are still challenges related to feedstock availability, reliable processing technologies, and higher biofuel costs. Recently, several studies reported on the feedstock availability, economic viability, and fuel compatibility of different biofuel options for marine applications. For example, Mukherjee *et al.*(10) analyzed and compared the performance and viability of biomass gasification, FT synthesis, hydrotreatment of yellow grease, and woody biomass fast pyrolysis (FP), as assessed by techno-economic analysis (TEA) and life cycle analysis. Along the same lines, comprehensive investigations were performed by the International Energy Agency (IEA) and by the Netherlands Maritime Knowledge Centre in 2017.^17^ Hansson *et al.*(7) used a different approach, examining the prospects of seven alternative marine fuels using multicriteria decision analysis and by interaction with Swedish stakeholders. The criteria used ranged from economic criteria such as fuel price and operational cost to environmental criteria such as acidification and health impact, thus providing a holistic picture of the merits and pitfalls of the fuels.

In this work, we conduct comparative TEA and environmental sustainability analysis for four biofuel production pathways for the marine sector, including (1) HTL of wet wastes such as sewage sludge and manure (pathway 1); (2) FP of woody biomass (pathway 2); (3) landfill gas FT synthesis (LGFT) (pathway 3); and (4) lignin–ethanol oil (LEO) pathway from a lignocellulosic ethanol biorefinery utilizing reductive catalytic fractionation (RCF) (pathway 4). The biofuels analyzed in this study are considered potential drop-in fuels or blendstocks compatible for use in marine engines; however, additional experimental work is needed to demonstrate the fuel compatibility with marine engines (*i.e.*, performance, reliability, and durability), meet the emission requirements, and evaluate the properties with respect to current standards. This analysis is based on the experimental data at small scales and rigorous process modeling for the scale-up of the selected pathways. This work adds to the existing scientific literature, including FT marine biofuels from the co-feeding of biomass with coal or natural gas and from yellow grease *via* the hydroprocessed ester and fatty acid process,^5^ by providing a comparative economic and sustainability assessment for four new pathways across multiple biomass feedstocks and processing options for potential marine biofuel applications. The comprehensive comparison aims to guide researchers and industry stakeholders on the potential opportunities and research needed for sustainable, low-cost biofuel options for the marine sector.

## 2 Methods and Assumptions

### 2.1 Process Models for Marine Biofuel Pathways

Process models for the selected marine biofuel pathways are developed for quantifying the process yield, raw materials, and energy consumption. Detailed process descriptions for the selected pathways are provided in the Supporting Information. Pathway 1 is wet waste HTL with two wet wastes (sewage sludge and manure). Plant scale is a key economic driver for this pathway, as shown in the sludge HTL design case.^18^ A preliminary wet waste resource analysis shows that 82% of the total wet waste resources in the United States could be collected at sites over a 1000 dry tonne/day scale at a transportation cost of $50/dry tonne (based on 2014 transportation costs).^19^ To take advantage of the economies of scale, a large HTL plant at a scale of 1000 dry tonne/day is modeled and evaluated. Pacific Northwest National Laboratory (PNNL) is currently investigating the potential of using raw, mildly hydrotreated, and fully hydrotreated biocrude for marine fuel or maritime fuel blends and the impact of biocrude properties and feedstock compositions on the viability of each of these options. To account for these three options being considered, three hydrotreatment scenarios are evaluated for each feedstock: no hydrotreatment, mild hydrotreatment, and full hydrotreatment. Figure S1 shows the process configuration used for this pathway as well as the potential minimum processing requirements for marine fuel, while Table S1 lists the key process variables of the HTL process for this analysis.

Pathway 2 includes FP-based processes. The pathway converts a 50/50 blend of forest residues and clean pine to bio-oil *via* three process options: FP without catalytic vapor upgrading (FP1) and FP with vapor phase upgrading over a ZSM-5 zeolite catalyst (FP2) and a Pt/TiO_2_ catalyst (FP3). Process flow diagrams are shown in Figure S2. All conceptual plant designs are based on a 2000 dry metric tonne per day feedstock rate. The process model for uncatalyzed FP (FP1) utilizes a circulating fluidized bed design. The dual-bed reactor system includes a riser reactor for FP and a char combustor to heat the circulating sand to maintain the reaction temperatures at 500 °C (932 °F) during pyrolysis.^20^ The majority of solids (including sand, char, and ash) are removed from the pyrolysis vapors *via* cyclones. In FP1, the pyrolysis vapors are condensed to produce bio-oil for potential use as a marine fuel. FP2 includes a subsequent *ex situ* catalytic fluidized reactor system for upgrading the pyrolysis vapors over a zeolite (ZSM-5) catalyst prior to the condensation step.^21^ FP3 also has subsequent *ex situ* vapor upgrading but uses a Pt/TiO_2_ catalyst in the fixed-bed parallel reactor system with online upgrading and offline regeneration operations. An additional hot gas filter is necessary for FP3 to remove any residual particulates in order to protect the fixed-bed system from plugging.^21,22^ The fluidized bed *ex situ* reactor in FP2 requires constant catalyst replenishment due to attrition losses in a circulating bed system; the fixed-bed design in FP3 does not require continuous catalyst replenishment, allowing the use of precious metal catalysts such as Pt/TiO_2_, and was shown to have higher yields of bio-oil.^23^ However, FP3 requires the introduction of hydrogen to promote yields, a potential operational safety concern; the FP2 fluidized *ex situ* reactor performance modeled here is based on experiments in 2016 that did not include the introduction of hydrogen,^24^ although hydrogen addition can be included as part of the design.^21^ It should be noted that while the detailed references provided here for pathway 2 will allow the reader to understand the process conversion configurations, the yields and configurations were adapted for this study, especially with the elimination of downstream hydrotreatment necessary for the near-complete deoxygenation of bio-oil required for standard terrestrial automobiles; complete deoxygenation is not a requirement for marine fuels. Although downstream hydrotreatment was eliminated, the catalytic steps in FP2 and FP3 yielded deoxygenated bio-oils, as tabulated in the results; the lower oxygen contents compared to that of FP1 correspond to more stable and less reactive/corrosive bio-oils. An additional aspect of FP3 is the recovery of valuable co-products, acetone and methyl-ethyl-ketone, which help lower the cost of the bio-oil. FP1 was modeled at a lower front-end pressure of 2.4 bar compared to the catalytic upgrading processes FP2 and FP3 modeled at 8.5 bar; the higher pressure helped to reduce the capital costs because of the smaller equipment volume.

Pathway 3 is a gas-to-liquid process that includes steam methane reforming (SMR), syngas conditioning (compression and acid gas removal), and FT synthesis (Figure S3, Supporting Information). The feedstock is landfill gas (LFG) instead of the more commonly used natural gas. LFG differs in the composition from that of natural gas, with approximately 40% of the volume as CO_2_. LFG comes off the header at the landfill at a pressure of 1.6 psig and must be compressed to the SMR operating pressure of 30 psi (2.1 bar). After compression, an iron bed removes H_2_S in the feed stream, followed by an activated carbon bed to remove any remaining siloxanes. With SMR, the primary reaction is to convert the methane gas to carbon monoxide and hydrogen (*i.e.*, syngas) with the injection of steam. Additionally, syngas and unreacted gases from the FT process may be recycled back to the reformer or combusted to provide some or all of the heat necessary for the endothermic reforming reactions. The syngas stream consisting mostly of CO, H_2_, H_2_O, and CO_2_ is cooled and compressed to 425 psi (29.3 bar) before entering the acid gas removal system, which removes the bulk of H_2_S and CO_2_ from the process gas. FT synthesis is a catalytic conversion process, which converts the synthesis gas to a mixture of reaction products, namely diesel- and gasoline-range synthetic fuels.^25^ The advantages of the FT polymerization process are that it offers the ability to produce liquid hydrocarbon fuels with a relatively low sulfur and aromatic content.^26^ The FT products are condensed and separated through a multicut distillation column to separate the product streams. The purified H_2_ from the PSA system is used for hydrotreating the distillation products to yield blendstocks for gasoline, diesel, and jet fuel or used for hydrocracking wax. Wax remaining after hydrocracking and the excess H_2_ not consumed during hydrotreating or hydrocracking are sold as co-products.

Pathway 4 is the LEO pathway. Figure S4 in the Supporting Information depicts the block flow diagram of an integrated biorefinery design. The LEO production conceptual process is similar to NREL’s 2011 cellulosic ethanol pathway studied for TEA.^27^ However, in the biomass pretreatment step, the dilute acid pretreatment was replaced by a reductive catalytic fractionation (RCF) process.^28^ Additionally, in this work, hybrid poplar instead of corn stover was used as the feedstock. Poplar is fast-growing and can be cultivated on marginal lands. Additionally, the energy crop exhibits a higher lignin content than herbaceous feedstocks and thus results in a higher LEO yield.^28^ The integrated biorefinery produces both ethanol and a depolymerized lignin-rich oil and allows for the integration of lignin–ethanol solvolysis.^28^ Biomass is fed with ethanol to RCF reactors (210 °C and 30 bar), selectively depolymerizing and reductively stabilizing lignin within the feedstock over a 5 wt % Pd/C catalyst to lignin-rich oil and carbohydrate-rich pulp. The former is LEO, and the latter is subsequently converted to ethanol *via* fermentation. The ethanol loading is set at 4.0 L/kg of dry biomass, and the residence time is 2 h (Table S3). While a portion of ethanol is degraded to CO and CO_2_ in the RCF reactor, a majority is recovered *via* distillation and recycled back to the reactor, resulting in a net ethanol consumption of approximately 60 g of ethanol/kg of LEO product. Ethanol produced from the biorefinery is supplied to the RCF reactor. The biomass delignification is 75%, which is mainly a mixture of monomeric lignin (10%), dimeric lignin species (35%), and oligomers (12%). The LEO’s lower heating value (LHV) is 21.6 MJ/kg. Natural gas is the supplemental fuel that is required for generating heat and power to meet the biorefinery demand. The pathway produces LEO and ethanol fuel products, and excess electricity is exported to the grid as a co-product.

### 2.2 Techno-economic Analysis

The TEA is performed based on mature or *n*th plant economic assumptions. Table S4 summarizes the primary financial parameter assumptions based on the U.S. Department of Energy’s Bioenergy Technologies Office guidelines.^22,29^ The *n*th plant method assumes that several plants have already been built and are operating successfully. Thus, this method does not account for special financing, equipment redundancies, large contingencies, and long startup times. The TEA model encompasses a process model and an economic model. The mass and energy balances for the selected processes can be solved with the detailed process model. Then, capital and operating costs, which were estimated from the mass and energy balances, are used in a discounted cash flow analysis to determine the minimum fuel selling price (MFSP) needed to meet a 10% internal rate of return when the net present value is set to zero. All costs are adjusted to 2016 U.S. dollars. The unit for the MFSP is dollars per HFO gallon equivalent (HFOGE). HFOGE is determined using eq 1, where the LHV basis for HFO (140,352 Btu/gal) is obtained from the GHGs, regulated emissions, and energy use in transportation model (GREET).^30^
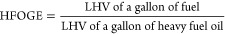
1

## 3 Results and Discussion

### 3.1 Process Performance

Table 1 summarizes the key process performance variables, such as fuel production, product yield, and fuel properties for all pathways. For the HTL pathway, the products include raw, mildly hydrotreated, and fully hydrotreated biocrude without further distillation. The product distribution is similar for all HTL scenarios, roughly 20% naphtha-range, 40% diesel-range, and 40% heavy residue fuels. The HTL pathway shows high HFOGE yield, carbon, and energy efficiencies. Carbon efficiency in this work is defined as the ratio of fuel carbon to feedstock carbon, while energy efficiency refers to the ratio of the fuel LHV to the sum of LHV of the feed and natural gas and electricity utilities. However, the process efficiencies decrease with the increasing hydrotreatment intensity due to the extra utilities and hydrogen for the hydrotreating steps. Also, manure HTL has relatively lower energy and carbon efficiencies due to high ash and oxygen contents in the feedstock. For the fuel properties, the S and O contents of the produced biofuel have a direct impact on the combustion emissions and marine fuel storage stability. Fuel heating value is another important factor for marine fuel. The fuel with a less heating value indicates lower energy density and thus can potentially harm the shipping’s economic operation. For the HTL pathways, the S content in the feedstocks has a direct impact on the S content in the produced biocrude. Although both raw biocrudes from the sludge and manure have >0.5% S content, partial or full hydrotreatment can effectively lower the S content. In addition, co-feeding with other non-sulfur or low-sulfur wet waste such as food waste, fat/oil/grease can provide another option to produce raw biocrude with a lower S content to meet the target with further hydrotreatment. Furthermore, the HTL-derived biofuel has a relatively lower O content and higher heating values.

**Table 1:** Key Process Performance Variables and Cost Worksheet (All the Costs Are in 2016 U.S. Dollars)^a^

pathways	sludge hydrothermal liquefaction (SHTL)			manure hydrothermal liquefaction (MHTL)			fast pyrolysis (FP)			landfill gas Fischer–Tropsch synthesis (LGFT)	lignin–ethanol oil
pathway index	SHTL1	SHTL2	SHTL3	MHTL1	MHTL2	MHTL3	FP1	FP2	FP3	LGFT	LEO
process performance:											
fuel production^b^	33.24	31.39	31.72	32.38	32.86	33.46	52.82	31.07	34.48	41.28	56.38
fuel yield^c^	107.76	101.74	102.83	101.36	106.52	108.46	72.94	42.90	47.61	n.a.^d^	77.86
energy efficiency (%)	71	67	63	66	61	58	64.7	38.0	42.2	52	47
carbon efficiency (%)	72	67	63	67	65	63	61.9	33.2	35.7	63	61
fuel properties:											
S content,wt %	1.11	0.39	0.00	0.70	0.24	0.01	0.00	0.00	0.00	0.00	0.00
O content,wt %	4.8	2.5	1.0	14.0	5.0	0.5	49.0	17.0	17.0	0.0	41.0
LHV (Btu/gal)	124,630	148,407	149,611	113,947	146,417	146,665	71,570	110,454	112,735	128,154	96,804
capital costs, $ million											
total installed cost (TIC)	94.14	128.46	157.22	94.11	130.15	160.94	209.16	217.77	282.78	274.10	338.95
fixed capital investment (FCI)	177.94	241.22	295.23	177.88	244.34	302.17	380.57	383.21	498.76	509.52	597.05
total capital investment (TCI)	187.68	255.30	313.07	187.62	258.62	320.50	401.45	404.22	522.55	536.61	628.75
total variable operating cost without feedstock credits	0.72	0.79	0.85	0.64	0.71	0.75	0.94	1.60	0.62	1.47	1.95
total variable operating cost with feedstock credits	–0.60	–0.62	–0.54	–0.17	–0.08	–0.03	0.94	1.60	0.62	1.47	1.95
fixed operating costs^e^	0.25	0.48	0.54	0.29	0.49	0.54	0.39	0.67	0.72	0.60	0.28
total operating cost without feedstock credits	1.27	1.34	1.40	0.93	1.20	1.29	1.33	2.27	1.34	2.07	2.23
total operating cost with feedstock credits	–0.05	–0.07	0.01	0.12	0.41	0.51	1.33	2.27	1.34	2.07	2.23

a Table S9 in the Supporting Information provides more detailed information on the capital and operating cost.

b In million HFOGE/year.

c In HFOGE/dry tonne biomass.

d Not available.

e General overhead equals 90% of total salaries; maintenance equals 3% of fixed capital investment; and insurance and taxes equal 0.7% of fixed capital investment.

The FP pathways (FP1-3) produce bio-oil, and the product yields (HFOGE/dry tonne) are dependent on the treatment of the pyrolysis vapor phase, with FP1 (no catalytic upgrading, 72.9) > FP3 (upgrading over the Pt/TiO_2_ catalyst, 47.6) > FP2 (upgrading over the ZSM-5 catalyst, 42.9). Note that the high yield in FP1 comes with the drawback of a higher O content and potential problems with stability. S and N contents are low in the FP cases using woody feedstocks. The LGFT pathway exhibits a yield of 41.3 million HFOGE/year (20% naphtha, 38% jet, and 43% diesel). The LEO pathway’s yield is 77.8 HFOGE/dry tonne (52% LEO and 48% ethanol). For all cases, the energy and carbon efficiencies correlate highly with the product yields.

### 3.2 Total Capital Investment

The capital investment for the four pathways is presented in Table 1, while the assumptions for capital cost estimates are provided in Table S5. For sludge HTL, the total capital investment (TCI) for the three scenarios increases in the order (in $ millions): SHTL1 (188) < SHTL2 (255) < SHTL3 (313). SHTL3 shows the highest TCI as it includes the hydrotreatment of guard bed and main bed reactors to fully remove the heteroatoms in the biocrude, and the substantial amount of hydrogen consumption requires a higher hydrogen plant cost. SHTL1 does not need additional capital cost associated with the hydrotreatment and hydrogen plant, while SHTL2 only includes a guard bed hydrotreatment reactor and a small amount of hydrogen for partially removing the O and S contents in the biocrude. The manure HTL exhibits a similar trend that the TCI increases with the hydrotreating severity, and the feedstock types have insignificant impact on TCI. The biocrude production step represents approximately 60% of the total installed equipment cost (TIC) for the fully hydrotreated scenario, while partially and fully hydrotreated steps contribute to 22 and 18% of the capital cost, respectively.

For FP pathways, the TCIs for FP1-3 are $401, $404, and $526 million, respectively. High TCI for FP3 is attributed primarily to the higher ancillary costs associated with hydrogen production and co-product purification; as noted previously, higher modeled pressures in FP2 and FP3 helped to reduce corresponding capital costs of the front-end pyrolysis equipment compared to that of FP1. The LGFT pathway costs $534 million, corresponding to the TCI-to-annual gallon of $11.87, which is similar to that of a gas-to-liquid plant reported in the literature.^31^ The cost of LEO pathway is the highest ($628 million) among all the pathways, where the high-pressure RCF alone contributes 40% of TIC.

### 3.3 Operating Costs

Operating costs, including labor costs, materials, and feedstock costs, utility costs, and disposal costs, were evaluated for all the pathways. Table S6 lists the detailed information for estimating the variable operating costs, including the catalysts, feedstocks, utilities, and disposal for the HTL pathways. Variable operating costs are determined based on the raw materials, waste-handling charges, and byproduct credits incurred only during the process operation. Fixed operating costs are generally incurred in full even if the plant is not operating at full capacity.^5^Table S9 demonstrates the breakdown of these operating costs and their contribution to the total production cost. For the HTL pathways, waste disposal cost is the biggest cost contributor and varies with the feedstock types. The waste disposal associated with the landfilling cost of solid wastes from the HTL and aqueous treatment contributes 30–40% of the total operating costs for the sludge and manure HTL. An average national landfill fee of $55.36/dry tonne was used in the analysis.^32^ Moreover, the operating costs increase by about $0.11/HFOGE for fully hydrotreating the sludge- or manure-derived biocrude. The potential feedstock credit for the avoided cost of disposal paid by wet waste generators can lower the fuel costs in the range of $0.78–$1.33/HFOGE, depending on the feedstock type and wet waste locations. The wet waste-avoided disposal cost is estimated based on the wet waste recourse analysis by Badgett *et al.*(33) and detailed in Tables S7 and S8. Feedstock costs represent the biggest cost driver for all non-HTL pathways: FPs (around 42%), LGFT (61%), and LEO (28%). The feedstock types and costs are FP pathways (50/50 blend of forest residues and clean pine, $70.15/dry tonne), LGFT (LFG, $3.70/MMBtu), and LEO (poplar, $80.00/dry ton). While LFG is assumed to be 70% the price of natural gas, the high specific feedstock cost associated with the LGFT pathway accounts for the high CO_2_ content. The normalized total operating costs that encompass the feedstock cost, co-product credits, and fixed operating costs range from $1.33/HFOGE (FP1) to $2.22/HFOGE (LEO).

### 3.4 Minimum Fuel Selling Price

**Figure 1:**
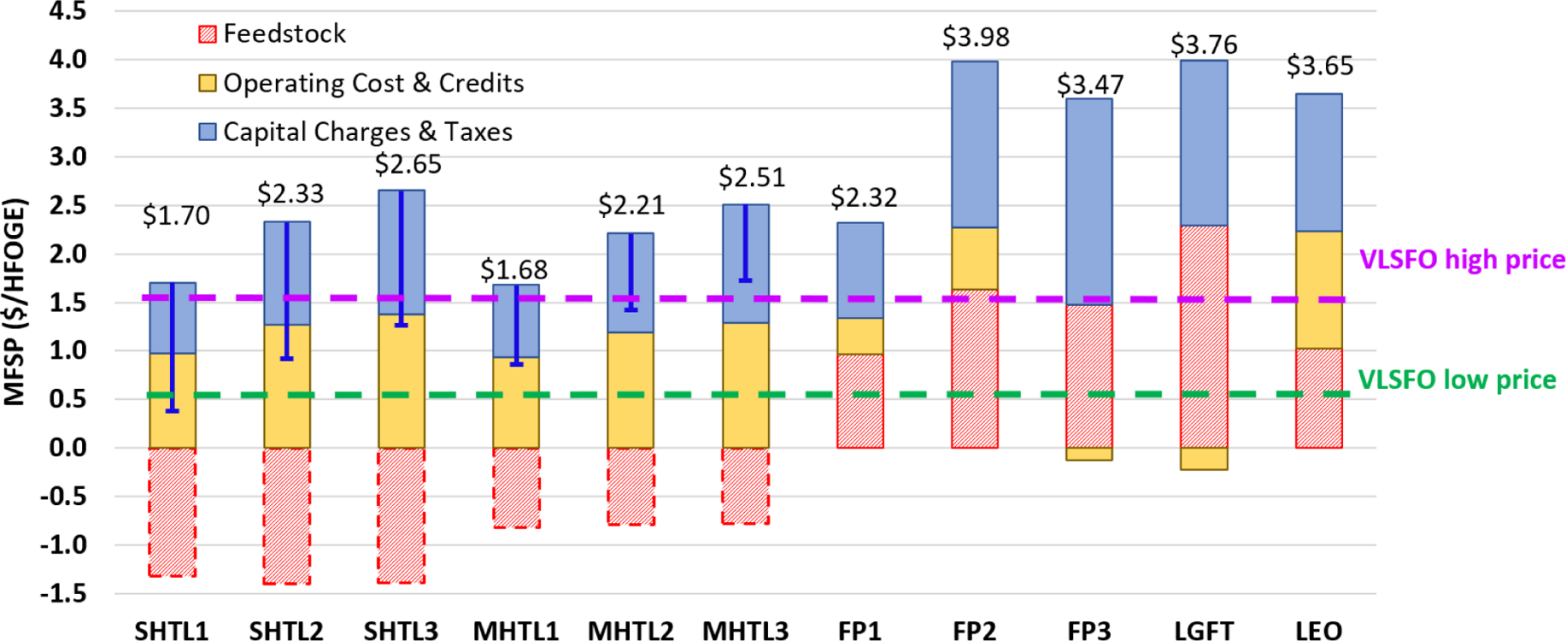
Comparative TEA result summary (the dash feedstock costs for HTL cases represent the sensitivity cases with the potential wet waste-avoided disposal fee, while the blue error bars indicate the potential decrease of MFSP for HTL pathways; high and low VLSFO prices are the last 2 years’ historical price range from the main ports of North America;^35^ see Table S10 in Supporting Information for biofuel prices from the literature).

Using the estimated plant capital and operating costs, a discounted cash flow rate of return calculation was performed to determine the MFSP that meets the economic parameter using the economic assumptions listed in Table S4. The fuel products are combined and collectively referred to as a single-fuel product on a HFOGE basis for simplicity. All MFSPs were determined and reported on a combined product basis. The cost contributions to the MFSP are divided into (i) capital charges and taxes, (ii) operating costs and co-product credits, and (iii) feedstock costs. MFSPs across the pathways range from $1.68 to $3.98 per HFOGE for the scenarios without feedstock credits (Figure 1).

MFSPs for the HTL pathways increase in the order: $1.68/HFOGE (MHTL1) < $1.70/HFOGE (SHTL1) < $2.21/HFOGE (MHTL2) < $2.33/HFOGE (SHTL2) < $2.51/HFOGE (MHTL3) < $2.65/HFOGE (SHTL3). The partially and fully hydrotreated processes increase the modeled MFSP for both wet wastes by about $0.58/HFOGE and $0.89/HFOGE, respectively, compared to the raw biocrude price. The wet waste cost is assumed to be zero in such analysis. Considering the potential avoided wet waste disposal fee involved in the current sludge and manure management, it is estimated that the average sludge and manure credits are $160/dry tonne and $125/dry tonne, respectively. Details of this calculation are presented in Tables S7 and S8. Note that the transportation costs for collecting 1000 dry tonne/day scale were also included in the analysis. The blue error bars in Figure 1 represent the impact of the potential feedstock credits on the MFSP by about −$1.40/HFOGE and −$0.79/HFOGE for sludge HTL and manure HTL, respectively.

Feedstock credits were not considered for non-HTL pathways as they do not apply to biomass feedstocks and LFG. MFSPs for non-HTL pathways are between $2.32/HFOGE (FP1) and $3.98/HFOGE (FP2). MSFPs for all non-HTL pathways except the LEO pathway are predominantly attributed to the feedstock (>40%). The LEO pathway’s cost distributions are capital charges and taxes (39%), operating costs and credits (33%), and feedstock (28%). The current TEA results were adopted to estimate the marginal abatement costs for life cycle emission reductions reported in the companion life cycle assessment paper.^34^

### 3.5 Sustainability Metrics for Conversion Plants

In addition to TEA performance, this work compares the process sustainability metrics (SMs) for the pathways considered, including biorefinery water intensity, wastewater generation, carbon conversion, and energy efficiencies, as well as biofuels’ sulfur and oxygen contents. The upstream and downstream processes, namely, feedstock production, fuel distribution, and fuel combustion are not within the scope of this analysis. Each process SM is normalized based on the maximum and minimum values of the selected pathways to facilitate the comparisons across all conversion pathways on a scale from 0 to 100%. Equation 2 shows the normalization formula
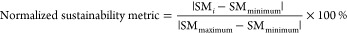
2

**Figure 2:**
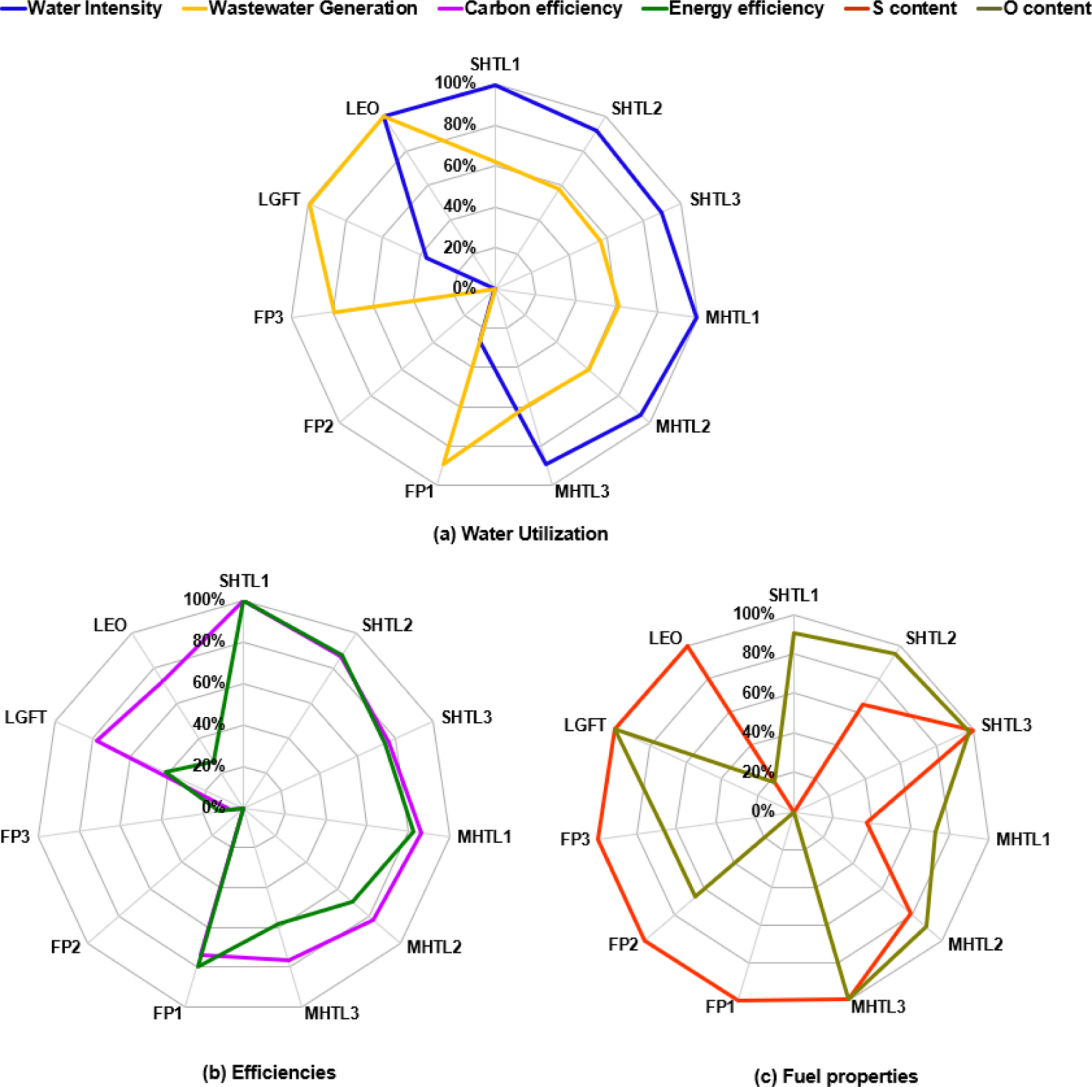
SM results (100 and 0% represent the best and worst case among all the pathways, respectively). The best–worst values for all the indicators: water intensity (0, 0.08 gal/MJ fuel), wastewater generation (0, 0.12 gal/MJ fuel), carbon efficiencies (33.2, 72.1%), S content (0, 1.11%), and O content (0, 49.5%).

Figure 2 shows the normalized SM for each pathway, while Table S12 in the Supporting Information gives each pathway’s actual SM values. Normalized SMs are binned into three impact categories: (a) water utilization, containing water consumption and wastewater generation, (b) carbon and energy efficiencies, and (c) fuel properties containing the sulfur and oxygen contents of the fuel (Figure 2). Water intensity and wastewater generation are important environmental metrics for biorefineries. The former is largely attributed to consumptive water usage, in which water is removed from the available supplies without returning to a water resource system, such as evaporation and drift at the cooling tower. The latter’s environmental footprints could include acidification and eutrophication that disrupt the natural balance of aquatic life. The water intensity for all the pathways ranges from 0.0004 to 0.08 gal/MJ fuel. As a reference, the refining process of crude oil to gasoline consumes between 0.02 and 0.06 gal/gasoline gallon equivalent. HTL and LEO pathways exhibit better water intensities than FP and LGFT pathways. Conversely, HTL pathways have low performance on wastewater generation as more than 98% of wastewater is from the wet wastes of high water content (75% moisture content in the feed) in the HTL processes.

Biomass carbon-to-fuel efficiency is also an important measurement of natural resource utilization and is inherent to biofuel sustainability. Both fuel yield and biomass carbon-to-fuel efficiency measure how efficient the technology is at producing the liquid fuel. The process efficiency also has a great impact on the process economic performance. As shown in Figure 2b, the energy and carbon efficiencies for HTL pathways are better than those for FP, LGFT, and LEO pathways. S and O contents in the produced biofuel can potentially harm the vessels’ heating system, make the fuel less stable, and increase polluting effects. Thus, S and O contents are considered an important sustainability factor during the fuel application stage. Figure 2c shows the S and O contents in the marine biofuel candidates. FP, LGFT, and LEO have nearly zero S content, with relatively moderate O content. In contrast, HTL-derived fuels show relatively higher S and O contents, but the O and S contents can significantly change depending on further treatment and feedstock composition.

### 3.6 Technical Feasibility

The current second-generation biofuels not derived from waste and nonfood feedstocks help overcome the constraints of first-generation biofuels that are mainly derived from food feedstock like corn and soybeans. Therefore, the second-generation biofuels alleviate competition with food production. Additionally, the second-generation biofuels using waste feedstock enable the bio-based circular carbon economy and help close the carbon cycle, stressing the opportunity to create an additional carbon sink capability in the technosphere by utilizing biogenic carbon for marine biofuels.^37^

Before any new fuel chemistry can be adopted for marine use, it must demonstrate compatibility with the existing fuel system infrastructure and suitable engine performance. If the fuel is to be introduced as a blend with HFOs, it must demonstrate miscibility and stability. To date, these types of studies have been very limited. The results are mixed. Some preliminary studies on raw FP bio-oils have shown good stability with some HFOs, while others have not.^36^ Likewise, the results are mixed for HTL biocrudes. However, stabilizing additives have shown the potential to improve the stability. Preliminary studies have also shown that low blend levels with HFOs exhibit suitable compatibility (based on corrosion and viscosity measurements) and combustion properties. The key findings are that the corrosivity of bio-oil becomes negligible for the blends with HFOs containing up to 50 wt % bio-oil and that the viscosity of HFOs is dramatically lowered by the low levels of bio-oil.

## Supporting Information Available

Detailed description of the process, TEA assumption, capital and operating cost estimation, wet waste feedstock cost estimation, and process performance summary (PDF)

## Abbreviations

FP: fast pyrolysis
FAME: fatty acid methyl ester
FOG: fat/oil/grease
FT: Fischer–Tropsch
GHG: greenhouse gas
HFO: heavy fuel oil
HFOGE: heavy fuel oil gallon equivalent
HTL: hydrothermal liquefaction
IEA: International Energy Agency
IMO: International Maritime Organization
LCA: life cycle analysis
LEO: lignin–ethanol oil
LGFT: landfill gas Fischer–Tropsch synthesis
LNG: liquefied natural gas
LHV: lower heating value
MFSP: minimum fuel selling price
MHTL: manure hydrothermal liquefaction
MGO: marine gas oil
PNNL: Pacific Northwest National Laboratory
RCF: reductive catalytic fractionation
SM: sustainability metric
SMR: steam methane reforming
SHTL: sludge hydrothermal liquefaction
TCI: total capital investment
TIC: total installed equipment cost
TEA: techno-economic analysis
VLSFO: very low sulfur fuel oil
NREL: National Renewable Energy Laboratory
