# Supplementary material for: Techno-economic Analysis of Sustainable Biofuels for Marine Transportation
Source: Environ Sci Technol. 2022 Nov 21;56(23):17206–14. doi: 10.1021/acs.est.2c03960 (PMC9730900; doi:10.1021/acs.est.2c03960)
Supplement: Supplementary file 1 — es2c03960_si_001.pdf [file es2c03960_si_001.pdf]

Supporting Information

Techno-economic Analysis of Sustainable Biofuels for Marine Transportation

Shuyun Li [a,e], Eric C. D. Tan [b,e,*], Abhijit Dutta [b], Lesley J Snowden-Swan [a], Michael R. Thorson [a], Karthikeyan K. Ramasamy [a,*], Andrew W. Bartling [b], Robert Brasington [b], Michael D. Kass [c], George G. Zaimes [d], Troy R. Hawkins [d]

[a] Pacific Northwest National Laboratory, Richland, WA 99352, USA

[b] National Renewable Energy laboratory, Golden, CO, 80401, USA

[c] Oak Ridge National Laboratory, Oak Ridge, TN 37830, USA

[d] Argonne National Laboratory, Lemont, IL 60439, USA

[e] These authors contributed equally.

* Corresponding authors:

eric.tan@nrel.gov

karthi@pnnl.gov

List of Figures

Figure S1. Wet waste HTL and biocrude upgrading process flow diagram. S4
Figure S2. Process flow diagram and process description of catalytic fact pyrolysis S6
Figure S3. Landfill gas Fischer-Tropsch synthesis block flow diagram.................................... S7
Figure S4. Simplified block flow diagram for the lignin-ethanol oil (LEO) pathway utilizing reductive catalytic fractionation.. S9

List of Tables

Table S1. Key assumptions for HTL conversion with two feedstocks S4
Table S2. Fuel composition for LFG compared to natural gas.. S8
Table S3. RCF reactor operating assumptions for lignin-ethanol oil production (Bartling et al., 2021)... S10
Table S4. Key assumptions for the techno-economic analysis... S11
Table S5. Cost factors for direct and indirect project costs... S12
Table S6. Variable operating costs for the HTL pathways. S13
Table S7. Data extracted from Badgett et al. (2019) for sludge feedstock cost (avoided disposal fee) estimate.. S14
Table S8. Data extracted from Badgett et al. (2019) for manure feedstock cost (avoided disposal fee) estimate.. S15
Table S9. Project cost worksheet (in 2016 $)... S16
Table S10. Potential marine fuel price in the open literature... S17
Table S11. Process performance summary. S18
Table S12. Sustainability metric values for the selected pathways. S18

S1. Process flow diagram and process description of wet waste HTL

As shown in Figure S1, the process begins with the high moisture content wet wastes collected from animal farms and water treatment plants. Prior to processing, the wet waste is dewatered to 25 wt% solids for minimizing the capital and operating cost of the HTL plant. In the base case, both two wet waste (sludge and manure) cost at the gate of the HTL plant is assumed to be zero. However, there exist potential significant savings in avoided disposal costs to farms and water treatment plants and the potential feedstock credits are detailed below. In addition, transportation cost for collecting wastes from multiple locations is considered to support the HTL plant scale. The HTL plant processes 1000 dry metric tons of wet waste a day and is briefly described below.

The HTL reactor operating condition is near the subcritical water status, which has a high solubility for organic compounds. To reach such operating conditions, the feed slurry is first pumped to 20 MPa and then heated in the heat exchanger and trim heater to reach 350 °C. The HTL reactor has a shell-and-tube structure, having feed slurry on the tube side while hot heating oil on the shell side. After the reaction, the wet waste is converted into biocrude, an aqueous phase, and a small number of solids and gases. In a solid-liquid-gas three-phase separator, solid and gas are separated from the liquid and then the liquid effluents are cooled for further aqueous-biocrude phase separation. The gas combined with natural gas is sent to a burner for generating heat to supply HTL heating requirements via the hot oil system. The aqueous phase needs a series of treatment steps before recycling back to the wastewater treatment (WWT) plant. Specifically, it is first treated with quicklime to raise the pH to ~11 and then stripped with air to remove ammonia and volatile organics (VOCs) from the aqueous stream. The ammonia and VOCs can be completely destroyed in a thermal oxidizer (THROX) with the help of natural gas and catalyst. At the same time, the liquid at the bottom of the stripper is further treated to decrease chemical oxygen demand (COD) before recycling back to WWT plants.

The produced biocrude is cooled to be used as marine fuel blendstock or can be further mildly- or fully-hydrotreated to improve the fuel properties. Specifically, the hydrotreating equipment includes a guard bed for metals and mild heteroatom removal and the main hydrotreating reactor for removing oxygen, nitrogen, and sulfur as much as possible. Different hydrotreating steps could be required for HTL biocrude from different wet wastes due to the variability of feedstock compositions. Figure S1 shows the potential minimum processing requirements for marine fuel

blendstocks. Note that the biocrude upgrading is co-located with the HTL plant in this scenario (i.e., no transportation cost for biocrude). Table S1 summarizes the key process technical assumptions associated with HTL of two feedstocks.

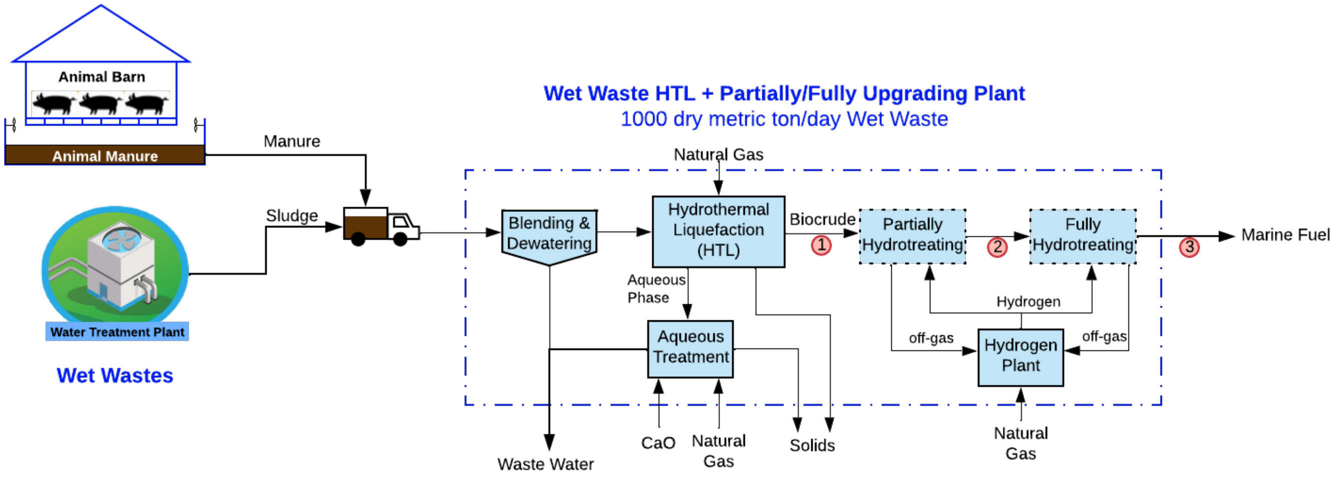

Figure S1. Wet waste HTL and biocrude upgrading process flow diagram.

Table S1. Key assumptions for HTL conversion with two feedstocks

	Sludge HTL	Manure HTL
HTL scale (dry tonne per day wet waste)	1,000	1,000
Upgrader scale (million gal/year biocrude)	37	40
Feedstock composition	50/50 primary/secondary Sludge	100% Manure
Feed solid (wt %)	25	25
Ash-free solid (wt %)	21	22
Biocrude yield (%)	44	50
Transportation cost of waste feedstock to HTL ($/dry tonne waste)	30	50
Transportation cost of biocrude ($/GGE [c])	0 (colocated with HTL)	0 (colocated with HTL)
Hydrotreating catalyst life (year)	1.0	0.5
H_2 consumption in guard bed (g/g dry feed)	0.027[a]	0.027[a]
H_2 consumption in main bed (g/g dry feed)	0.019[b]	0.016[b]

[a] an average H_2 consumption in the guard bed is based on the data with the H_2 consumption range of 0.007-0.035 g/g dry feed for algae HTL at different hydrotreating conditions.
[b] is based on sludge HTL and manure HTL operating data.
[c] gasoline gallon equivalent

S2. Process flow diagram and process description of catalytic fast pyrolysis

As discussed in the manuscript, three process options were considered for evaluating the minimum fuel selling prices (MFSP) for fast pyrolysis systems. Configuration FP1 (Figure S2a) is the simplest, without the inclusion of (i) catalytic upgrading, (ii) hydrogen use, and (iii) chemical coproducts recovery. It is also the case with the highest yield, but at the cost of a poorer product quality (high O content in the liquid fuel/intermediate usually renders it reactive and unstable during long term storage). All the FP process configurations include the production of electricity from pyrolysis off-gases and any excess heat available from char combustion. The FP1 pathway is near commercial for pyrolysis-oil production, and technology offerings are available for different fast pyrolysis reactor configurations.

Configuration FP2 (Figure S2b) includes an *ex situ* catalytic reactor following the fast pyrolysis reactor for upgrading the fast pyrolysis vapors from the fast pyrolysis reactor. The upgrading reactor design in FP2 is a circulating fluidized system with a zeolite catalyst. Hydrogen is not added to the system. The catalytic upgrading reduces the O content of the product fuel and makes it more stable for handling and downstream upgrading.

Configuration FP3 (Figure S2c) is the most complex among the three configurations (Dutta et al., 2021). In addition to a fixed bed *ex situ* upgrading reactor with Pt/TiO_2 catalyst, it includes the use of hydrogen (generated from process off-gases) to boost yields. Recovery of oxygenated coproducts acetone and methyl-ethyl-ketone (MEK) are also included; nearly 4.3% of the biomass carbon is retained in the recovered coproducts, providing a high value revenue complement for the fuel product, and helps drive the net operating cost negative (FP3 in Figure 1); the coproducts also help improve the sustainability metrics and GHG emissions for the process, when accounted using a product displacement method.

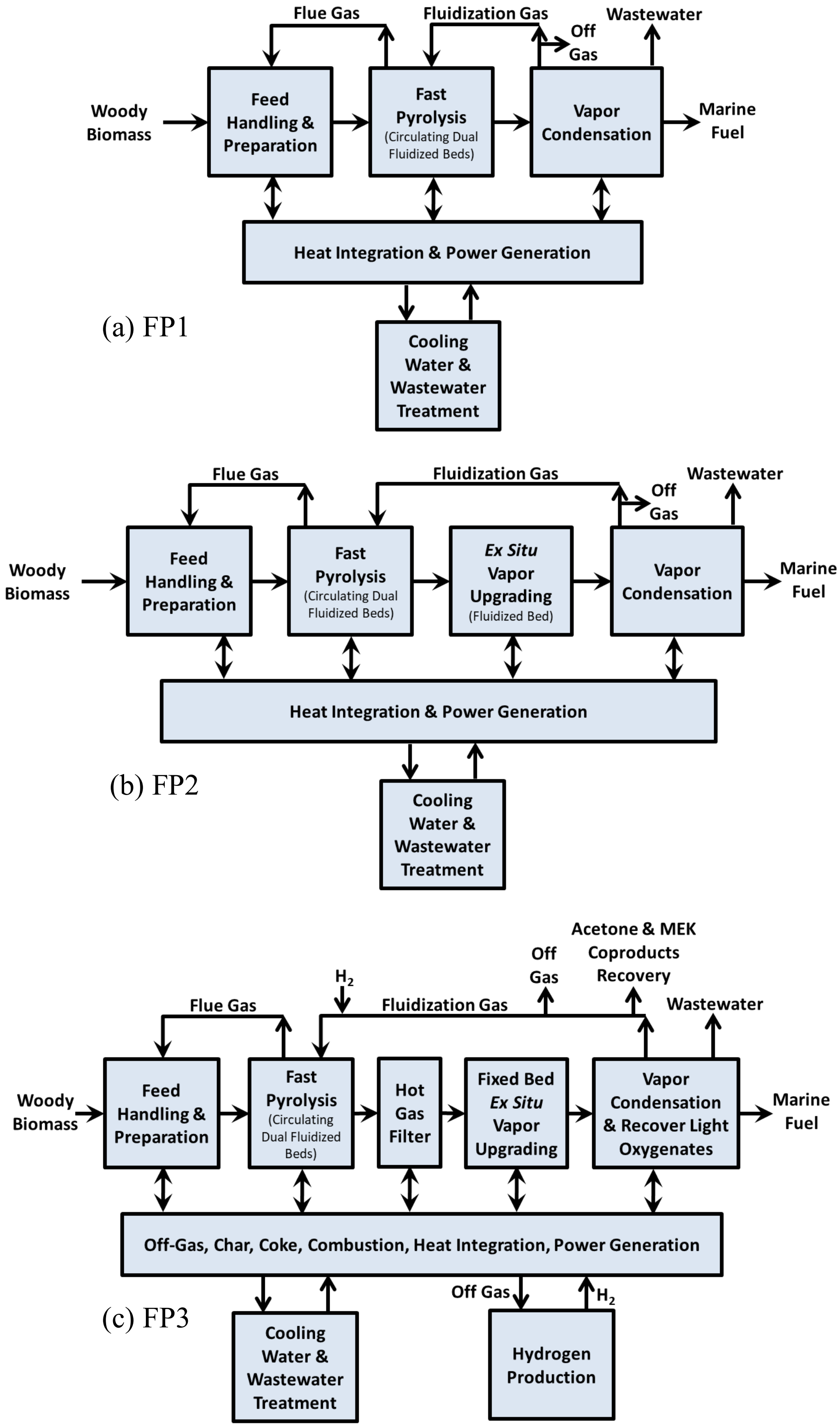

Figure S2. Process flow diagram and process description of fast pyrolysis (a) fast pyrolysis (FP1), (b) catalytic fast pyrolysis with ZSM-5 catalyst in a fluidized bed (FP2), (c) catalytic fast pyrolysis with Pt/TiO_2 catalyst in a fixed bed (FP3).

S3. Process flow diagram and process description of landfill gas Fischer-Tropsch synthesis

Figure S3 depicts the simplified process flow diagram of the landfill gas Fischer-Tropsch synthesis process. The feedstock is landfill gas (LFG) instead of the more commonly used natural gas. LFG differs in composition from natural gas, with approximately 40 percent of the volume as CO_2. A compositional breakdown is provided in Table S2. LFG comes off of the header at the landfill at a pressure of 1.6 psig and must be compressed to the steam reforming operating pressure of 30 psi (2.1 bar). After compression, an iron bed removes the H_2S in the feed stream, followed by an activated carbon bed to remove any remaining siloxanes.

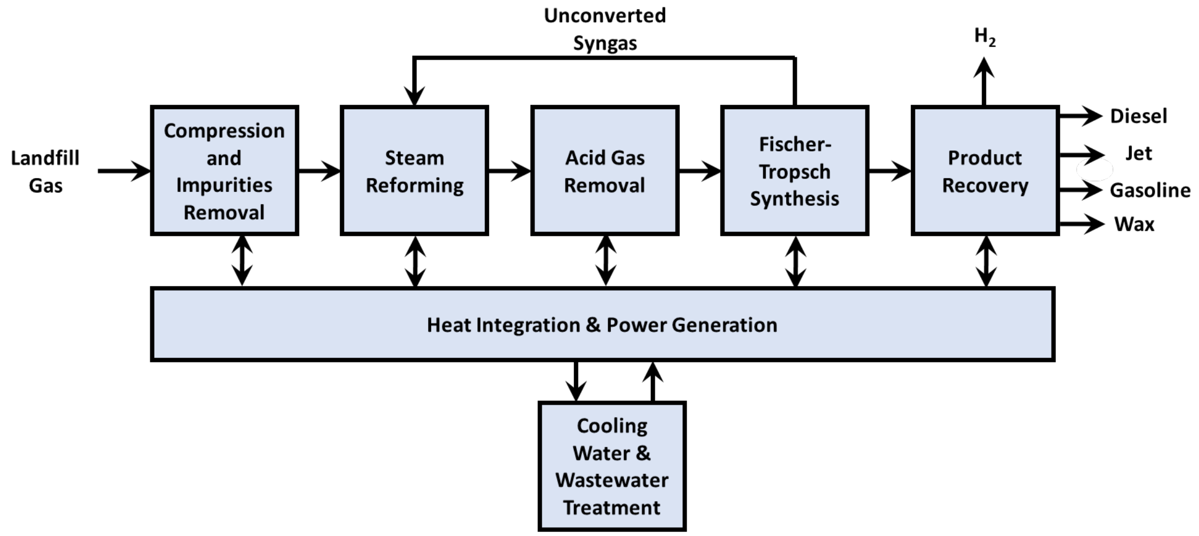

Figure S3. Landfill gas Fischer-Tropsch synthesis block flow diagram.

Table S2. Fuel composition for LFG compared to natural gas.

	Landfill gas	Natural gas
Constituents	Concentration (vol%)	
Methane (CH_4)	57.1	94.4
Ethane (C_2H_6)	0	3.1
Propane (C_3H_8)	0	0.1
Isobutane (i-C_4H_{10})	0	0.5
N-butane (n-C_4H_{10})	0	0.1
Isopentane (i-C_5H_{12})	0	0.2
Carbon monoxide (CO)	6 ppm	0
Nitrogen (N_2)	2.4	1.1
Carbon dioxide (CO_2)	40.5	0.5
Hydrogen sulfide (H_2S)	68 ppm	6 ppm
Siloxanes	6 ppm	0

Steam reforming, i.e., $CH_4 + H_2O \rightarrow CO + 3H_2$, converts methane gas to carbon monoxide and hydrogen. Additionally, unconverted syngas from the FT is partly recycled back to the reformer and partly combusted to provide the heat necessary for the endothermic reforming reactions. The syngas stream, consisting mostly of CO, H_2, H_2O, and CO_2, is cooled and then compressed to 425 psi (29.3 bar) before entering the acid gas removal system, which removes the bulk of the H_2S and CO_2 from the process gas.

Fischer-Tropsch (FT) synthesis is a catalytic conversion process, which converts the synthesis gas to a mixture of reaction products such as diesel, gasoline, jet fuel, and wax products. The overall reaction involved in the FT synthesis is represented with $(2n + 1)H_2 + nCO \rightarrow C_nH_{2n+2} + nH_2O$. The advantages of the FT polymerization process are that it offers the ability to produce liquid hydrocarbon fuels with relatively low sulfur and aromatic content. For this process, the H_2/CO ratio entering the FT reactor was maintained at 1.5:1 by sending a portion of the synthesis gas stream to a pressure swing adsorption (PSA) system with the offgas feeding the FT reactor. The impact of the lower H_2/CO ratio results in a less than stoichiometric ratio being fed to the reactor, and thus only 78 percent of the CO is consumed in the FT reactor, compared to a typical natural gas feedstock which has an 85 percent conversion of CO.

The FT products are condensed and separated through a multi-cut distillation column to separate the product streams. The purified H_2 from the PSA system is used for hydrotreating distillation products to yield blendstocks for gasoline, diesel, and jet-fuel or used for hydrocracking wax. Wax

produced from the hydrocracker is sold as a co-product. Any excess H_2 not consumed for hydrotreating or hydrocracking is sold as a co-product.

S4. Process flow diagram and process description of lignin-ethanol oil

Figure S4. illustrates the block flow diagram of a reactive reductive catalytic fractionation (RCF) based cellulosic ethanol biorefinery that co-produces lignin ethanol oil (LEO) according to Bartling et al. (2021). This process's configuration is similar to that described by Humbird et al., (2011), with the dilute-acid pretreatment area replaced with RCF. The RCF area produces a lignin-rich oil, here also containing a small fraction of residual ethanol (i.e., LEO) as a co-product and a carbohydrate-rich pulp. The latter is the residual biomass solids after the lignin is removed during RCF and is isolated and saccharified to C5 and C6 sugars via enzymatic hydrolysis, fermented to ethanol, and recovered to produce fuel-grade ethanol. A combination of natural gas, sludge from wastewater treatment, and residual solids from ethanol production are burned to generate steam for process heat and electricity via a combined heat and power (CHP). Excess electricity is sold to the grid as a co-product. The RCF reactor operating assumptions for lignin-ethanol oil production are summarized in Table S3.

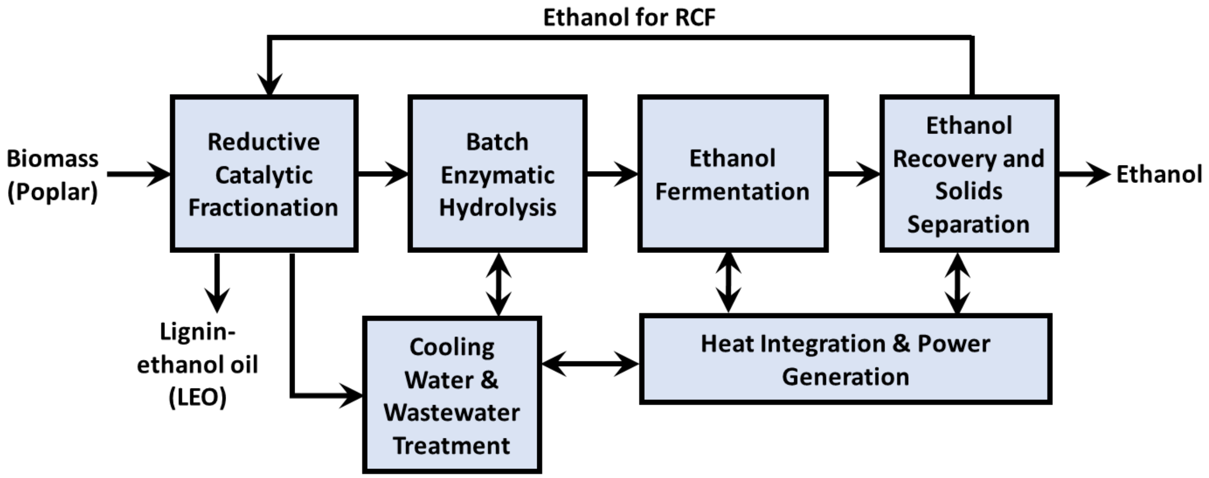

Figure S4. Simplified block flow diagram for the lignin-ethanol oil (LEO) pathway utilizing reductive catalytic fractionation (RCF).

Table S3. RCF reactor operating assumptions for lignin-ethanol oil production (Bartling et al., 2021).

Reactor solvent (volumetric ratio)	1:1 ethanol: water
Solvent loading (L per dry kg biomass feed)	4.0
Temperature (ºC)	210
Pressure (bar)	30
Residence time (h)	2
Catalyst	5 wt% Pd/C
Biomass delignification (wt%)	75%
Solubilized lignin composition (wt%)	
Monomers	20%
Dimers	60%
Oligomers	20%
S-Monomer composition (wt%)	
4-Propylsyringol	75%
Dihydrosinapyl alcohol	25%
G-Monomer composition (wt%)	
4-Propylguaiacol	66%
Dihydroconiferyl alcohol	34%
Carbohydrate retention (wt%)	
Cellulose	97%
Xylan	38%
Arabinan	8%
Galactan	38%
Mannan	38%
Ethanol reforming to gases (wt% of ethanol)	0.50%

S5. Techno-economic Analysis Key Assumptions

Table S4 summarizes the primary economic assumptions for the "nth-plant" method employed in this study. This method does not account for special financing, equipment redundancies, large contingencies, and long startup times since it assumes several plants have already been built and are operating.

Table S4. Key assumptions for the techno-economic analysis.

Assumption Description	Assumed Value
Cost year	2016 $
Internal rate of return (IRR)	10%
Plant financing debt/equity	60% / 40% of total capital investment (TCI)
Plant life	30 years
Income tax rate	21%
Interest rate for debt financing	8.0% annually
Term for debt financing	10 years
Working capital cost	5.0% of fixed capital investment (excluding land)
Depreciation schedule	7-years MACRS schedule [a]
Construction period	3 years (8% 1st yr, 60% 2nd yr, 32% 3rd yr)
Plant salvage value	No value
Start-up time	6 months
Revenue and costs during start-up	Revenue = 50% of normal<br>Variable costs = 75% of normal<br>Fixed costs = 100% of normal
On-stream factor	90% (7,920 operating hours per year)

[a] Modified accelerated cost recovery system

S6. Capital cost estimates assumptions

HTL Pathway capital costs for each plant area are based on data from wet waste HTL design reports and publications from the Pacific Northwest National Laboratory (Li et al., 2021; Snowden-Swan et al., 2021). The assumptions of total direct cost, fixed capital investment, and total capital investment estimation are listed in Table S5 for all the pathways.

Table S5. Cost factors for direct and indirect project costs.

Direct Costs	
**Item**	**% of Total Installed Cost (TIC)**
Buildings	4.0%
Site development	10.0%
Additional piping	4.5%
Total Direct Costs (TDC)	18.5%
**Indirect Costs**	
**Item**	**% of TDC**
Prorated expenses	10%
Home office & construction fees	20%
Field expenses	10%
Project contingency	10%
Startup and permits	10%
Total Indirect Costs	60%
Working Capital	5% of Fixed Capital Investment (FCI)
Land	HTL: 6 acres @ $15,000/acre<br>Upgrading: 6% of Total Purchased Equipment Cost

S7. Variable Operating Costs

Table S6 lists the raw material, waste disposal, and utility costs assumed for estimating variable operating costs for the HTL plant.

Table S6. Variable operating costs for the HTL pathways.

Variable Operating Costs	Value	Reference
Raw Materials		
Polymer, $lb (2013$)	1.73	City of Detroit 2014
Quicklime, $/ton (2014$)	118	USGS 2016
Hydrotreating guard bed catalyst, $/lb (2014$)	16.6	IHS 2014c
Hydrotreating main bed catalyst, $/lb (2014$)	10.5	IHS 2014c
Hydrogen plant catalyst, $/1000scf H2 (2014$)	0.0205	IHS 2014b
Cooling tower chemical, $/lb (2007$)	1.36	Humbird et al. 2011
Boiler chemical, $/lb (2007$)	2.27	Humbird et al. 2011
Natural Gas, $/1000 scf (2016$)	3.51	EIA 2016a
Electricity, ¢/kWh (2016$)	6.76	EIA 2016b
Solid waste disposal fee, $/US ton (2019$)	55.36	EREF 2019
Wastewater POTW fee, $/tonne (2001$)	0.53	Dutta et al 2011

Sewage sludge management cost account for 40-50% of a wastewater treatment facility's total annual operating cost and includes dewatering/drying cost, and tipping fees paid to final use/disposal facilities. National Renewable Energy Laboratory's wet waste resources analysis shows that 64% of sludge in the US has a negative price and the average sludge price is $-36/wet ton (not including the dewatering cost) (Badgett et al., 2019), as shown in Table S7. Bay Area Clean Water Agencies (BACWA) has collected biosolid information from 31 wastewater treatment plants every two years and tracked down the most current industry trends on the sludge management options and the tipping costs. BACWA 2021 biosolids survey reports sludge cost range of $14-139/ wet ton with an average of $52/wet ton for the Bay Area (Bay Area Clean Water Agencies, 2021). A similar cost range of $21-102/wet ton is reported based on the data collected at Tohopekaliga Water Authority (FL), City of Calgary, City of Orlando, Metro Vancouver, and Santa Rosa Water (Marrone, 2016). In this work, $36/wet ton ($160/dry ton) (not including dewatering cost) is a realistic average value representative of what POTWs currently would pay for the offtake of their waste solids. In addition, capital and operating costs associated with polymer and electricity consumption for sludge dewatering are included in the HTL plant costs.

Table S7. Data extracted from Badgett et al. (2019) for sludge feedstock cost (avoided disposal fee) estimate.

Cumulative Percent	Wet Sludge Supply, millions of tonne	Sludge Price, $/wet tonne
0.0%	0.0	-152.9
2.8%	2.3	-123.5
8.8%	7.1	-120.6
15.5%	12.5	-113.2
21.8%	17.6	-102.9
24.4%	19.7	-69.1
28.1%	22.6	-58.8
33.0%	26.6	-51.5
38.2%	30.7	-48.5
42.8%	34.5	-39.7
47.2%	38.1	-42.6
53.5%	43.1	-29.4
59.3%	47.8	-22.1
66.7%	53.7	-5.9
74.0%	59.7	0.0
81.3%	65.5	2.9
88.5%	71.3	2.9
95.0%	76.6	7.4
100.0%	80.6	8.8
Weighted Average Cost, $/wet tonne		-40
Weighted Average Cost, $/wet ton		-36

Manure management cost includes on-farm nutrient management costs, off-farm transport costs, land treatment costs, manure and wastewater handling storage costs, and recordkeeping cost. National Renewable Energy Laboratory's wet waste resources analysis (Badgett et al., 2019) shows that 27% of animal manure in the US has a negative price and the average sludge price is $-48/wet ton ($-125/dry ton), as shown in Table S8. A wide range of $5 - 150/dry ton is reported as the swine manure avoided disposal cost based on the U.S. Department of Agriculture (USDA) method for estimating comprehensive nutrient management plans (CNMP) cost (Ou et al., 2022).

Table S8. Data extracted from Badgett et al. (2019) for manure feedstock cost (avoided disposal fee) estimate.

Cumulative Percent	Wet Manure Supply, millions of tonne	Manure Price, $/wet tonne
0%	9.5	-310.7
3%	12.4	-276.7
4%	14.9	-256.6
5%	15.6	-216.4
5%	16.0	-173.6
7%	17.9	-123.3
10%	21.9	-119.5
11%	22.7	-100.6
13%	25.3	-90.6
15%	28.0	-88.1
17%	30.9	-73.0
20%	35.3	-73.0
23%	39.4	-67.9
27%	43.6	-65.4
30%	48.1	-64.2
33%	51.7	-57.9
36%	55.3	-52.8
39%	59.8	-49.1
43%	64.6	-47.8
46%	69.4	-44.0
50%	74.0	-40.3
54%	78.7	-35.2
58%	83.9	-32.7
62%	89.5	-28.9
66%	94.4	-28.9
69%	98.9	-27.7
73%	104.0	-22.6
76%	108.0	-18.9
80%	112.5	-18.9
83%	116.5	-15.1
86%	121.0	-15.1
89%	125.2	-13.8
93%	129.7	-12.6
96%	134.2	-3.8
98%	137.0	6.3
100%	139.1	7.5
Weighted Average Cost, $/wet tonne		-53
Weighted Average Cost, $/wet ton		-48

Table S9. Project cost worksheet (in 2016 $)

		SHTL1	SHTL2	SHTL3	MHTL1	MHTL2	MHTL3	FP1	FP2	FP3	LGFT	LEO
**Capital Costs, $ million**												
**Total Purchased Equipment Cost (TPEC)**		**52.41**	**71.80**	**89.57**	**52.30**	**72.49**	**91.82**	**85.34**	**90.97**	**140.94**	**121.26**	**212.74**
Installed Factor		1.80	1.79	1.79	1.80	1.80	1.75	2.45	2.39	2.01	2.26	1.59
**Total Installed Cost (TIC)**		**94.14**	**128.46**	**157.22**	**94.11**	**130.15**	**160.94**	**209.16**	**217.77**	**282.78**	**274.10**	**338.95**
**Other Direct Cost (ODC)**												
Building	4%TIC	3.77	5.14	6.29	3.76	5.21	6.44	6.20	4.70	6.26	9.59	7.82
Site development	10%TIC	9.41	12.85	15.72	9.41	13.02	16.09	15.51	11.75	15.65	23.97	17.59
Add'l piping	4.5% ISBL	3.89	4.31	5.29	3.89	4.34	5.38	6.98	5.29	7.04	10.79	8.80
**Total Direct Cost (TDC)**	TIC+ODC	**111.21**	**150.76**	**184.52**	**111.18**	**152.71**	**188.85**	**237.86**	**239.51**	**311.72**	**318.45**	**373.16**
**Indirect Costs (IC)**	60%TDC	**66.73**	**90.46**	**110.71**	**66.71**	**91.63**	**113.31**	**142.71**	**143.71**	**187.03**	**191.07**	**223.89**
**Fixed Capital Investment (FCI)**	TDC+IC	**177.94**	**241.22**	**295.23**	**177.88**	**244.34**	**302.17**	**380.57**	**383.21**	**498.76**	**509.52**	**597.05**
Working Capital (WC)	5%FCI	8.90	12.06	14.76	8.89	12.22	15.11	19.03	19.16	24.94	25.48	29.85
Land (LC)		0.85	2.01	3.08	0.85	2.06	3.22	1.85	1.85	1.85	1.61	1.85
**Total capital investment (TCI)**	FCI+WC+LC	**187.68**	**255.30**	**313.07**	**187.62**	**258.62**	**320.50**	**401.45**	**404.22**	**525.55**	**536.61**	**628.75**
**TCI/TPEC**		**3.58**	**3.56**	**3.50**	**3.59**	**3.57**	**3.49**	**4.70**	**4.45**	**3.74**	**4.43**	**2.96**
**Operating Costs, $/HFOGE**												
Feedstock		0.00	0.00	0.00	0.00	0.00	0.00	0.96	1.64	1.47	2.29	1.03
Potential feedstock credits		-1.33	-1.40	-1.39	-0.82	-0.79	-0.78	0.00	0.00	0.00	0.00	0.00
Natural gas		0.07	0.08	0.12	0.09	0.14	0.18	0.00	0.00	0.00	0	0.58
Catalyst, chemicals etc.		0.16	0.18	0.19	0.14	0.15	0.15	0.02	0.63	0.06	0.15	0.53
Electricity		0.09	0.10	0.11	0.15	0.16	0.16	0.00	0.00	0.00	0.00	0.00
Waste disposal		0.43	0.43	0.43	0.27	0.26	0.26	0.02	0.08	0.04	0.0001	0.01
Co-product credits		0.00	0.00	0.00	0.00	0.00	0.00	-0.06	-0.74	-0.95	-0.97	-0.19
**Total variable operating cost without feedstock credits**		**0.72**	**0.79**	**0.85**	**0.64**	**0.71**	**0.75**	**0.94**	**1.60**	**0.62**	**1.47**	**1.95**
**Total variable operating cost with feedstock credits**		**-0.60**	**-0.62**	**-0.54**	**-0.17**	**-0.08**	**-0.03**	**0.94**	**1.60**	**0.62**	**1.47**	**1.95**
**Fixed operating costs***		**0.25**	**0.48**	**0.54**	**0.29**	**0.49**	**0.54**	**0.39**	**0.67**	**0.72**	**0.60**	**0.28**
**Total operating cost without feedstock credits**		**1.27**	**1.34**	**1.40**	**0.93**	**1.20**	**1.29**	**1.33**	**2.27**	**1.34**	**2.07**	**2.23**
**Total operating cost with feedstock credits**		**-0.05**	**-0.07**	**0.01**	**0.12**	**0.41**	**0.51**	**1.33**	**2.27**	**1.34**	**2.07**	**2.23**
MFSP without feedstock credits		1.70	2.33	2.65	1.68	2.21	2.51	2.32	3.98	3.47	3.76	3.65
MFSP with feedstock credits		0.38	0.92	1.26	0.86	1.42	1.73	2.32	3.98	3.47	3.76	3.65

* General overhead equals 90% of total salaries, maintenance equals 3% of fixed capital investment, and insurance and taxes equal 0.7% of fixed capital investment.

Table S10. Potential marine fuel price in the open literature.

Pathways	MFSP range, $/HFOGE	Feedstocks	Reference
Fischer–Tropsch	4.58	biomass and coal	
Fischer–Tropsch	3.62	biomass	
Fischer–Tropsch	2.87	biomass and natural gas	
Fischer–Tropsch	2.36	natural gas	(Tan et al., 2021)
biodiesel via hydrotreating	4.23	yellow grease	
biodiesel via hydrotreating	3.57	yellow grease + heavy fuel oil	
fast pyrolysis	2.36	low-ash woody feedstock	
decentralized Fischer–Tropsch	8.88	biomass	
centralized Fischer–Tropsch	3.70	biomass	(Carvalho et al., 2021)
fermentation	6.22	biomass	
pyrolysis	4.59	biomass	
gasification to liquid	7.34	bagasse	
hydrothermal liquefaction	3.71	bagasse	(Tanzer et al., 2019)
pyrolysis	4.50	bagasse	

Table S11. Process performance summary.

	SHTL1	SHTL2	SHTL3	MHTL1	MHTL2	MHTL3	FP1	FP2	FP3	LGFT	LEO
Fuel Production [1]	33.24	31.39	31.72	32.38	32.86	33.46	52.82	31.07	34.48	41.28	56.38
Fuel Yield [2]	107.76	101.74	102.83	101.36	106.52	108.46	72.94	42.90	47.61	n.a.[3]	77.86
Energy Efficiency	71%	67%	63%	66%	61%	58%	64.7%	38.0%	42.2%	52%	47%
Carbon Efficiency	72%	67%	63%	67%	65%	63%	61.9%	33.2%	35.7%	63%	61%
Fuel Properties:											
Density, g/ml	0.98	0.96	0.96	0.96	0.96	0.96	1.20	0.97	0.97	0.83	1.25
Carbon Content	75%	85%	86%	72%	84%	85%	44%	76%	75%	82%	54%
S, wt%	1.11	0.39	0.00	0.70	0.24	0.01	0.00	0.00	0.00	0.00	0.00
LHV (Btu/gal)	124630	148407	149611	113947	146417	146665	71570	110454	112735	128154	96804

Note: [1] in MMHFOGE/yr; [2] in HFOGE/dry ton biomass; [3] not available.

Table S12. Sustainability metric values for the selected pathways.

Indicators Values	SHTL1	SHTL2	SHTL3	MHTL1	MHTL2	MHTL3	FP1	FP2	FP3	LGFT	LEO
Fossil energy consumption, MJ/ MJ fuel	0.14	0.15	0.23	0.16	0.25	0.33	0.10	0.10	0.05	0.00	0.20
Water Intensity (makeup),[1] gal/MJ fuel	0.000	0.006	0.009	0.000	0.005	0.008	0.059	0.080	0.080	0.051	0.000
Wastewater Generation, gal/MJ fuel	0.047	0.053	0.054	0.049	0.049	0.051	0.013	0.125	0.026	0.001	0.000
Carbon efficiency, %	72%	67%	63%	67%	65%	63%	62%	33%	36%	63%	61%
Energy efficiency, %	72%	67%	63%	66%	61%	58%	65%	38%	42%	52%	47%
S, wt%	1.11	0.39	0.00	0.70	0.24	0.01	0.00	0.00	0.00	0.00	0.00
O, %	4.8%	2.5%	1.0%	14.0%	5.0%	0.5%	49.0%	17.0%	17.0%	0.0%	41.0%

Note: [1] Makeup water may be further reduced by recycling generated wastewater after additional treatment.

Reference

Badgett, A., Newes, E., Milbrandt, A., 2019. Economic analysis of wet waste-to-energy resources in the United States. Energy 176, 224–234. https://doi.org/10.1016/j.energy.2019.03.188

Bartling, A.W., Stone, M.L., Hanes, R.J., Bhatt, A., Zhang, Y., Biddy, M.J., Davis, R., Kruger, J.S., Thornburg, N.E., Luterbacher, J.S., Rinaldi, R., Samec, J.S.M., Sels, B.F., Román-Leshkov, Y., Beckham, G.T., 2021. Techno-economic analysis and life cycle assessment of a biorefinery utilizing reductive catalytic fractionation. Energy Environ. Sci. 14, 4147–4168. https://doi.org/10.1039/D1EE01642C

Bay Area Clean Water Agencies, 2021. Bay Area Clean Water Agencies 2021 Biosolids Trends Survey Report.

Carvalho, F., Portugal-pereira, J., Junginger, M., Szklo, A., 2021. Biofuels for maritime transportation: A spatial, techno-economic, and logistic analysis in brazil, europe, south africa, and the usa. Energies 14. https://doi.org/10.3390/en14164980

Dutta, A., Mukarakate, C., Iisa, K., Wang, H., Talmadge, M., Santosa, D., Harris, K., Baddour, F., Hartley, D., Cai, H., Ou, L., Schaidle, J., Griffin, M., 2021. Ex Situ Catalytic Fast Pyrolysis of Lignocellulosic Biomass to Hydrocarbon Fuels: 2020 State of Technology. Golden, CO (United States). https://doi.org/10.2172/1805204

Humbird, D., Davis, R., Tao, L., Kinchin, C., Hsu, D., Aden, A., Schoen, P., Lukas, J., Olthof, B., Worley, M., Sexton, D., Dudgeon, D., 2011. Process Design and Economics for Biochemical Conversion of Lignocellulosic Biomass to Ethanol: Dilute-Acid Pretreatment and Enzymatic Hydrolysis of Corn Stover. Golden, CO (United States). https://doi.org/10.2172/1013269

Li, S., Jiang, Y., Snowden-Swan, L.J., Askander, J.A., Schmidt, A.J., Billing, J.M., 2021. Techno-economic uncertainty analysis of wet waste-to-biocrude via hydrothermal liquefaction. Appl. Energy 283, 116340. https://doi.org/10.1016/j.apenergy.2020.116340

Marrone, P., 2016. Genifuel Hydrothermal Processing Bench-Scale Technology Evaluation Report. IWA Publishing.

Ou, L., Li, S., Tao, L., Phillips, S., Hawkins, T., Singh, A., Snowden-Swan, L., Cai, H., 2022. Techno-economic Analysis and Life-Cycle Analysis of Renewable Diesel Fuels Produced with Waste Feedstocks. ACS Sustain. Chem. Eng. 10, 382–393. https://doi.org/10.1021/acssuschemeng.1c06561

Snowden-Swan, L., Billing, J., Thorson, M., Schmidt, A., Jiang, Y., Santosa, D., Seiple, T., Daniel, R., Burns, C.A., Li, S., Hart, T., Fox, S., Olarte, M., Kallupalayam Ramasamy, K., Anderson, D., Hallen, R., Radovcich, S., Mathias, P., Taylor, M., 2021. Wet Waste Hydrothermal Liquefaction and Biocrude Upgrading to Hydrocarbon Fuels: 2020 State of Technology, Pacific Northwest National Laboratory. Richland, WA (United States). https://doi.org/10.2172/1771363

Tan, E.C.D., Hawkins, T.R., Lee, U., Tao, L., Meyer, P.A., Wang, M., Thompson, T., 2021. Biofuel Options for Marine Applications: Technoeconomic and Life-Cycle Analyses. Environ. Sci. Technol. 55, 7561–7570. https://doi.org/10.1021/acs.est.0c06141

Tanzer, S.E., Posada, J., Geraedts, S., Ramírez, A., 2019. Lignocellulosic marine biofuel: Technoeconomic and environmental assessment for production in Brazil and Sweden. J. Clean. Prod. 239, 117845. https://doi.org/10.1016/j.jclepro.2019.117845
